# Functions and Mechanisms of Lysine Glutarylation in Eukaryotes

**DOI:** 10.3389/fcell.2021.667684

**Published:** 2021-06-24

**Authors:** Longxiang Xie, Yafei Xiao, Fucheng Meng, Yongqiang Li, Zhenyu Shi, Keli Qian

**Affiliations:** ^1^Institute of Biomedical Informatics, Cell Signal Transduction Laboratory, Bioinformatics Center, Henan Provincial Engineering Center for Tumor Molecular Medicine, School of Basic Medical Sciences, Huaihe Hospital, Henan University, Kaifeng, China; ^2^Infection Control Department, The First Affiliated Hospital of Chongqing Medical University, Chongqing, China

**Keywords:** glutarylation, SIRT5, glutaryl-CoA, PTM, proteomic

## Abstract

Lysine glutarylation (Kglu) is a newly discovered post-translational modification (PTM), which is considered to be reversible, dynamic, and conserved in prokaryotes and eukaryotes. Recent developments in the identification of Kglu by mass spectrometry have shown that Kglu is mainly involved in the regulation of metabolism, oxidative damage, chromatin dynamics and is associated with various diseases. In this review, we firstly summarize the development history of glutarylation, the biochemical processes of glutarylation and deglutarylation. Then we focus on the pathophysiological functions such as glutaric acidemia 1, asthenospermia, etc. Finally, the current computational tools for predicting glutarylation sites are discussed. These emerging findings point to new functions for lysine glutarylation and related enzymes, and also highlight the mechanisms by which glutarylation regulates diverse cellular processes.

## Introduction

Protein post-translational modifications (PTMs), covalent chemical modifications of amino acid residues (Macek et al., 2019), are a conserved mechanism adopted by organisms to effectively modulate biological activities, enabling them to make rapid adaptive responses to environmental changes (Bernal et al., 2014). PTMs have been reported to be involved in various biological processes (Walsh et al., 2005; Xu et al., 2016). They play crucial roles in the diversification of protein functions in different biological and physiological interactions (Walsh et al., 2005; Xu et al., 2016). To date, 676 different PTMs have been identified in the UniProt database^1^, including lysine (Lys) acylation, phosphorylation, ubiquitination, SUMOylation, and so forth (Venne et al., 2014; Harmel and Fiedler, 2018). Lysine acylation is a widely occurring PTM of proteins (Diallo et al., 2019). Besides the well-known acetylation (Kac) (Kim et al., 2006; Choudhary et al., 2009; Zhao et al., 2010; Lundby et al., 2012), eight types of short-chain Lys acylations have recently been identified on histones, including Lys propionylation (Kpr) (Chen et al., 2007), butyrylation (Kbu) (Chen et al., 2007), 2-hydroxyisobutyrylation (Khib) (Dai et al., 2014), succinylation (Ksucc) (Xie et al., 2012), malonylation (Kma) (Xie et al., 2012), glutarylation (Kglu) (Tan et al., 2014), crotonylation (Kcr) (Tan et al., 2011), and β-hydroxybutyrylation (Kbhb) (Xie Z. et al., 2016). These modifications are similar to the well-studied Kac in their ε-amine linkage, but different in hydrocarbon chain length, hydrophobicity or charge (Sabari et al., 2017). Such as, compared to the Kac changing the charge of lysine from +1 to 0 and adding a 2-carbon acyl group to lysine (Hirschey and Zhao, 2015), Kglu means adding glutaryl groups to specific lysine residues (Dou et al., 2021). Kglu changes the charge of lysine from +1 to −1 and adds a 5-carbon acyl group to lysine (Hirschey and Zhao, 2015). These changes may lead to structural alterations on proteins, affecting their physiological functions and disrupting any interactions between the lysine side chains of glutarylated proteins and negative charged molecules (Hirschey and Zhao, 2015).

The Kglu was first identified by Tan et al. (2014). They performed immunoblot analysis with whole-cell lysates from *Escherichia coli*, *Saccharomyces cerevisiae*, Drosophila melanogaster (S2), mouse (MEFs), and human cells (HeLa). The results showed that Kglu is a conserved PTM and exists in both eukaryotic and prokaryotic cells. Afterward, more proteins including histone and non-histone proteins were identified as glutarylated proteins, and they were found to play important roles in mitochondrial functions (Schmiesing et al., 2018), oxidative damage (Zhou et al., 2016), sperm motility (Cheng et al., 2019), and glutaric aciduria 1 (GA1) (Dimitrov et al., 2020).

**FIGURE 1:**
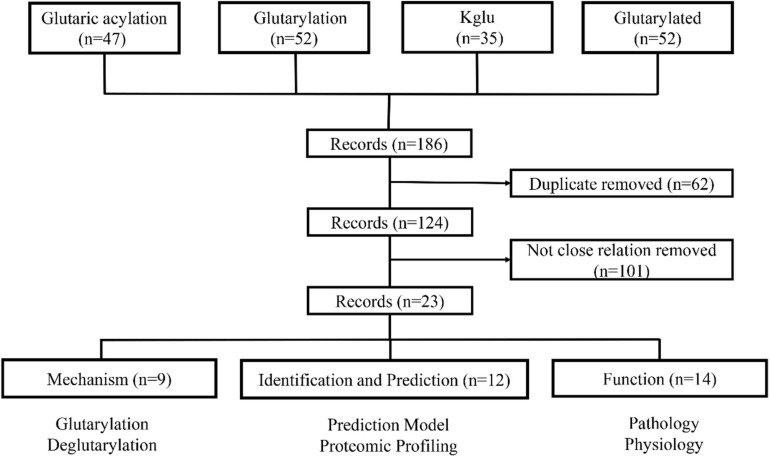
The process of literature screening. The articles were sorted into three categories: mechanism, function, identification and prediction of glutarylated proteins. *N* = number of literature records.

To systematically review the roles of Kglu in prokaryotes and eukaryotes, we searched PubMed for studies that mentioned glutarylation. Our specific advanced search terms included: “Glutaric acylation” OR “glutarylation” OR “Kglu” OR “glutarylated.” The results provided 186 papers as of January 10, 2021. Two investigators reviewed each initial study to determine whether it was related with glutarylation. Finally, 23 papers were preserved by exclusion criteria described in Figure 1. In this review, we mainly summarized recent studies about Kglu and discussed its implications. The scope mainly includes mechanism, function, identification and prediction of glutarylated proteins.

## Proteomic Profiling of Lysine Glutarylation

**FIGURE 2:**
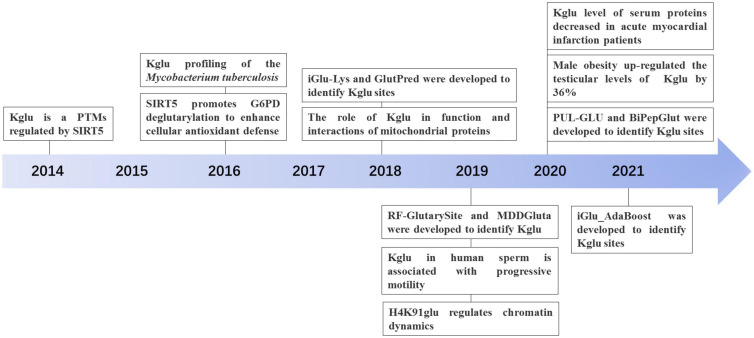
The discovery and development history of Lysine glutarylation (Kglu).

With the development of proteomic technology, the landscape of glutarylation is expanding (Figure 2). The proteomic method combing the sensitive immune-affinity purification and high-resolution liquid chromatography-tandem mass spectrometry (LC-MS/MS) has been used to find new glutarylated proteins and modification sites. Recently, four studies have identified new glutarylated proteins and glutarylated lysine residues in *Mycobacterium tuberculosis*, mouse and human serum. Xie L. et al. (2016), our group identified a total of 24 glutarylated proteins and 41 Kglu sites in *M. tuberculosis*. Schmiesing et al. (2018) found 37 glutarylated proteins with 73 Kglu sites in the brain of mice. Bao et al. (2019) revealed that Kglu occurs at 27 lysine residues on human core histones. Zhou et al. (2020) reported 4 kinds of glutarylated proteins with 10 sites in human serum. It shows that Kglu occurred in different species, and most of glutarylated proteins contain at least two Kglu sites in Table 1.

**TABLE 1:** Information on glutarylated proteins and sites that have been found.

**Time (year)**	**Experimental subject**	**Position**		**Protein (n)**	**Sites (n)**	**References**
		**Organ**	**Ultra structure**			
2014	*E. coli*	-	-	13	23	Tan et al., 2014
2014	HeLa cell	-	-	10	10	Tan et al., 2014
2014	Mouse	liver	-	191	683	Tan et al., 2014
2014	Mouse	liver	nucleus	1 (H2B)	3	Tan et al., 2014
2016	*Mycobacterium tuberculosis*	-	-	24	41	Xie L. et al., 2016
2018	Mouse	brain	-	37	73	Schmiesing et al., 2018
2018	Mouse	liver	-	154	425	Schmiesing et al., 2018
2019	HeLa cell	-	nucleus	4	27	Bao et al., 2019
2020	Human serum	-	-	4	13	Zhou et al., 2020
2020	Rat serum	-	-	2	4	Zhou et al., 2020

Since the discovery of immonium ion on acetylated lysine, most of the modifications based on acylation have their corresponding immonium ion characteristics. Parameter 86 m/z was set for detecting this PTM after MS/MS searches.

Antibodies are crucial for experimental studies, especially for the identification of protein PTMs. Therefore, we have reviewed the experimental studies on antibody for lysine glutarylation, and found eight related articles (Tan et al., 2014; Xie L. et al., 2016; Zhou et al., 2016, 2020; Schmiesing et al., 2018; Bao et al., 2019; Cheng et al., 2019; Wang et al., 2020). The specific Methods and reagents used in previous studies were summarized in Supplementary Table 1. Among eight studies, the glutarylation antibodies used in five studies are from the same company BioLabs (Tan et al., 2014; Xie L. et al., 2016; Zhou et al., 2016; Schmiesing et al., 2018; Cheng et al., 2019). Tan et al. (2014) found that the number of glutarylated peptides is 157, and the number of unmodified peptides is 297. Meanwhile, they performed Dot-blot assay using anti-Kglu antibody by incubation of the peptide libraries bearing a fixed unmodified lysine (K), acetyl-lysine (Kac), malonyl-lysine (Kmal), succinyl-lysine (Ksucc), glutaryl-lysine (Kglu), respectively. Each peptide library includes 10 residues CXXXXKXXXX, where X is a mixture of 19 amino acids (excluding cysteine), C is cysteine, and the 6th residue is a fixed lysine residue. The results showed that only peptide bearing a fixed Kglu can be detected, whereas other types of peptides cannot be detected. This shows the specificity of this glutarylation antibody (Tan et al., 2014). For the remaining three studies, Bao et al. (2019) raised a rabbit polyclonal antibody which is site specific against histones glutarylation and conducted dot-blot and western blot analysis to verify whether the antibody has high specificity. Although the origin of the antibodies was not specified in the remaining two papers, western blot analysis or mass spectrometer were also performed to verify the specificity of antibodies (Wang et al., 2020; Zhou et al., 2020).

Anyway, proteomic profiling of Kglu provides a key resource for finding novel properties and regulatory functions of Kglu.

## The Discovery of Lysine Glutarylation

Tan et al. (2014) first identified and validated Kglu as a PTM by four independent approaches, which is present in both prokaryotes and eukaryotes. They not only first discovered the presence of Kglu in *E. coli*, but also detected 23 Kglu sites in 13 glutarylated proteins. In addition, they isolated 10 glutarylated proteins in HeLa cells and detected 10 Kglu sites, and they also detected 683 sites in 191 glutarylated proteins in mouse liver cells. They also demonstrated that Kglu could be regulated by sirtuin 5 (SIRT5) and nutrient and showed that glutaryl-CoA could directly lead to non-enzymatic Kglu. They further showed that carbamoyl phosphate synthase (1), as a glutarylated protein, is associated with glutaric academic type 1 (GA1). Furthermore, they identified three Kglu sites on core histone H2B (H2BK5, H2BK116, and H2BK120), which are critical for regulation of gene expression (Tan et al., 2014). This research is significant, and it opens the door for further biological studies of Kglu (Figure 2).

## Lysine Glutarylation and Deglutarylation

**FIGURE 3:**
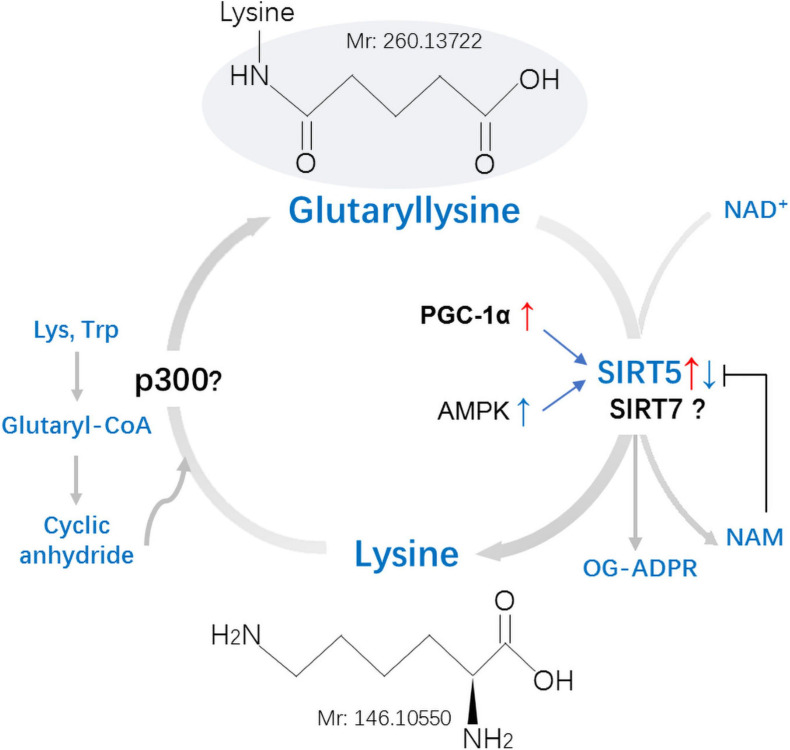
Mechanisms and regulation of non-histones lysine glutarylation (Kglu). Glutaryl-CoA forms a reactive cyclic anhydride that readily glutarylates lysine residues on target proteins. No enzymes were found in this process *in vivo*. Whether the p300 is involved in the process of Kglu remains to be identified. Kglu is targeted for removal by the NAD^+^-dependent SIRT5. Expression of SIRT5 can be inhibited by NAM and be regulated by PGC-1α and AMPK. Whether the SIRT7 possesses potent deglutarylase activities remains to be verified *in vivo*. Lys, Lysine; Trp, Tryptophan; NAM, Nicotinamide; OG-ADPR, O-Glutaryl ADP-Ribose; PGC-1α, peroxisome proliferator-activated receptor coactivator-1α; AMPK, AMP-activated protein kinase; Mr, molecular mass.

Glutaryl coenzyme A (CoA) serves as the main acyl donor molecule for Kglu reaction (Schmiesing et al., 2018). SIRT5, which relies on nicotinamide adenine dinucleotide (NAD^+^), can catalyze lysine deglutarylation *in vivo* and *in vitro* (Schmiesing et al., 2018). Glutarylation was considered a non-enzymatic process in the past (Tan et al., 2014), but in recent years, it has been found that glutarylation can be achieved enzymatically in histones (Bao et al., 2019; Figure 3).

### Lysine Glutarylation

Non-enzymatic reactions: Similar to acetyl coenzyme A and succinyl coenzyme A, glutaryl-CoA can directly induce the non-enzymatic Kglu (Tan et al., 2014). Cellular glutaryl-CoA forms a reactive cyclic anhydride that readily glutarylates lysine residues (Harmel and Fiedler, 2018). The level of Kglu is affected by multiple factors: (1) decreasing the concentration of other CoAs, thus reducing the competition with glutaryl-CoA; (2) increasing the concentration of glutaryl-CoA, both means could enhance the level of Kglu *in vivo* (Sabari et al., 2017).

Enzymatic reaction: P300, a member of histone acetyltransferases (HATs), is a well-studied transcription co-activator (Sabari et al., 2017). Apart from its initially described acetyltransferase activity (Bannister and Kouzarides, 1996; Ogryzko et al., 1996), P300 was also found to catalyze the Ksucc (Sabari et al., 2017) and Kglu (Tan et al., 2014) of histone. Although P300 has enzymatic activity for the modification of Kglu *in vitro*, no acyltransferase could catalyze the transfer of malonyl, succinyl, or glutaryl to proteins *in vivo* (Tan et al., 2014). Bao et al. (2019) found that KAT2A (lysine acetyltransferase 2A) and α-ketoadipate dehydrogenase (α-KADH) complex were conjugated, which could play the role of histone glutamyltransferase in cells. This implies that both P300 and KAT2A may be the glutaryl transferase responsible for the histone glutarylation. It will be very interesting to study whether these two glutaryl transferases also can catalyze the glutarylation of non-histone proteins in the cytoplasm and mitochondria.

### Lysine Deglutarylation

Sirtuins are a class of protein deacylases and/or ADP ribosyltransferases that depend on NAD^+^ (Kumar and Lombard, 2018). In mammals, the sirtuin family consists of seven members (sirt1–7) that have conserved NAD^+^ binding and catalytic domains (Kumar and Lombard, 2017). Zhao et al. (2010) proved that the previously annotated deacetylase SIRT5 is a lysine depentadiene Chemase (Tan et al., 2014). One recent research showed that SIRT5 is mainly present in mitochondria, cytoplasm, and nuclear loci (Park et al., 2013). SIRT5 is involved in glycolysis, tricarboxylic acid (TCA) cycle, fatty acid oxidation, and reactive oxygen species (ROS) detoxification (Kumar and Lombard, 2018).

Kglu is a PTM regulated by SIRT5, which possesses potent desuccinylase, demalonylase, and deglutarylase activities (Tan et al., 2014). At physiological pH, succinyl, malonyl and glutaryl will negatively charge the modified lysine residue (Hirschey and Zhao, 2015). There are two positively charged amino acid groups in the active center of SIRT5 (Du et al., 2011; Peng et al., 2011; Zhou et al., 2012). Therefore, it is not difficult to understand that SIRT5 displays a unique affinity for negatively charged acetyllysine modification and catalyzes protein desuccinylation, demalonylation, and deglutarylation. The SIRT5-catalyzed deglutarylation reaction requires NAD^+^ as a cofactor, which is inhibited by nicotinamide, a class III HDAC inhibitor (Tan et al., 2014). Expression of SIRT5 can be regulated by peroxisome proliferator-activated receptor coactivator-1α (PGC-1α) and AMP-activated protein kinase (AMPK) (Buler et al., 2014). Overexpression of PGC-1α increased SIRT5 mRNA and protein levels, whereas AMPK overexpression inhibited SIRT5 expression in primary mouse hepatocytes (Buler et al., 2014). Under normal basal conditions, the depletion of SIRT5 does not result in an indispensable effect on cell metabolism (Osborne et al., 2016).

Another study showed that SIRT7 catalyzed the hydrolysis of glutaryl peptides in the presence of nicotinamide adenine dinucleotide (NAD) and DNA *in vitro* and in cells (Bao et al., 2019). This implies that SIRT7 may be a compensatory mechanistic pathway. However, whether the SIRT7 possesses potent deglutarylase activities remains to be verified *in vivo*.

## Functional Roles of Lysine Glutarylation

### Regulation of Metabolism

Glutaryl-CoA, one of the precursors of glutarylation, is a thiol ester compound of glutaric acid and coenzyme A (Menon and Stern, 1960; Nishizuka et al., 1960). Glutaric acid, derived from lysine and tryptophan, is mainly metabolized in mitochondria, and the metabolism of glutaryl-CoA is also mainly located in the mitochondria (Besrat et al., 1969; Vamecq et al., 1985). Glutaryl-CoA dehydrogenase (GCDH) is a key enzyme in the metabolic process of glutaryl-CoA (Cheng et al., 2019). GCDH catalyzes the oxidative decarboxylation of glutaryl-CoA to crotonyl-CoA in the lysine and tryptophan degradation pathways, and the increase of glutaryl-CoA content in GCDH KO mice elevated the level of Kglu (Goodman and Frerman, 1995; Koeller et al., 2002; Tan et al., 2014).

It has been discovered that protein glutaryl metabolism mainly occurs in mitochondria (Schmiesing et al., 2018). For example, proteomic analysis of mouse liver revealed that there are 191 glutarylated proteins, of which 148 are mainly or partly located in mitochondria, accounting for more than three-quarters of all identified glutarylated proteins (Tan et al., 2014). There are two reasons why glutaryl metabolism mainly exists in mitochondria: It may be because glutaryl-CoA is mainly located in mitochondria (Besrat et al., 1969; Vamecq et al., 1985); in addition, this may be related to the higher pH (7.9) of mitochondrial matrix which is associated with the deprotonation of the ε-amino group of lysine, making them more susceptible to acylation (Carrico et al., 2018). Mitochondria play a key role in energy production, cell signaling and cell survival, and mitochondrial dysfunction can lead to the aging and aging-related diseases, such as metabolic diseases, cancer, and neurodegeneration (Osborne et al., 2016). Since acyl-CoA cannot penetrate the inner mitochondrial membrane, its accumulation in the mitochondrial compartment is easy. Accumulation of toxic acyl-CoA will affect mitochondrial energy metabolism (Dimitrov et al., 2020). Glutaryl-CoA inhibits the E2 subunit of α-ketoglutarate dehydrogenase complex (KGDHc), similar to the feedback inhibition of its physiological product, succinyl-CoA, leading to mitochondrial TCA cycle dysfunction (Sauer et al., 2005). Notably, the reduction of α-ketoglutarate dehydrogenase (KGDH) activity has recently been demonstrated in other neurodegenerative diseases, such as Alzheimer (Gibson et al., 1988), Parkinson (Mizuno et al., 1994, 1995), and Huntington diseases (Klivenyi et al., 2004), sharing neuropathological similarities with GCDH deficiency (Strauss and Morton, 2003).

In addition, lysine glutarylation can also affect mitochondrial metabolism and other mitochondrial functions (Ju and He, 2018). CPS1, mainly found in mitochondria (Summar et al., 1995), is the first rate-limiting enzyme in the urea cycle (UC), which is responsible for directly incorporating ammonia into the intermediate of UC (Nitzahn and Lipshutz, 2020). It is verified that CPS1 is a substrate of Kglu, and that Kglu of CPS1 inhibits its enzymatic activity (Tan et al., 2014). Excessive glutarylation will reduce the activity of CPS1 enzyme, resulting in increased blood ammonia levels and damage to nerve cells (Nakagawa et al., 2009; Tan et al., 2014; Nitzahn and Lipshutz, 2020).

### Regulation of Asthenospermia

Unlike somatic cells, mature sperm has a highly concentrated chromatin structure. Except for a few genes that are expressed in sperm mitochondria, there is almost no transcription and translation activities (Gur and Breitbart, 2008). Therefore, compared with somatic cells, the function regulation of mature sperm cells is more dependent on PTMs (Cheng et al., 2019).

The most widespread PTM in human sperm studies is phosphorylation, followed by acetylation, and these modifications are essential for sperm differentiation, maturation, and function (Porambo et al., 2012; Yu et al., 2015). Cheng et al. (2019), for the first time, reported the cofactor and regulatory protein of human sperm Kglu, and also discussed the correlation between sperm Kglu and sperm motility, as well as the role of Kglu in asthenospermia. As the energy metabolism center, mitochondria are vital to sperm motility (Yu et al., 2015). They found that Kglu is clearly present in the mitochondria of normal male sperm, while the content of Kglu is reduced in weak sperm (Bakos et al., 2011). Therefore, they speculated that Kglu is involved in the regulation of human sperm mitochondrial function. The decrease of Kglu in mitochondria may damage mitochondrial function and ultimately affect sperm motility (Cheng et al., 2019).

In addition, many studies have proven that obesity can reduce sperm quality and functions, which cause sperm DNA damage, and lead to hypogonadism (Jensen et al., 2004; Ghanayem et al., 2010; MacDonald et al., 2010; Bakos et al., 2011; Dupont et al., 2013; Samavat et al., 2014; Ramaraju et al., 2018). Wang et al. (2020) found that obesity in men can increase testicular histone Kglu levels by 36%. Although they proved that Kglu is related to male reproductive dysfunction caused by obesity, whether there is a causal relationship between increased histone Kglu and decreased sperm motility still needs to be verified.

### Regulation of Oxidative Stress

It has been reported that Kglu is closely related to oxidative metabolism (Tan et al., 2014; Zhou et al., 2016; Chen et al., 2020). Tan et al. (2014) through GO enrichment analysis, found that Kglu was significantly enriched in many cellular metabolic processes, including redox and aerobic respiration. They also found the potential impact of Kglu on metabolic pathways of oxidative metabolism through KEGG and Pfam enrichment analysis (Tan et al., 2014). Study has showed that reactive oxygen regulatory proteins such as superoxidase dismutase could be a substrate of the enzymes involved in the addition of glutarylation (Xie L. et al., 2016).

The central nervous system has a high metal content, which can catalyze the formation of oxygen free radicals, and its antioxidant defense ability is relatively low, so it is vulnerable to free radical damage (Facheris et al., 2004). It is known that excessive mitochondrial ROS are the main cause of cellular oxidative stress (Tojyo et al., 1989; Turrens, 2003; Orrenius et al., 2007; Park et al., 2011). GSH can remove ROS and protect cells from oxidative damage (Zhou et al., 2016). Nicotinamide Adenine Dinucleotide Phosphate (NADPH) is the main intracellular reducing agent and plays a key role in keeping glutathione in its reduced form of GSH. Zhou et al. found that deglutarylation can activate one NADPH-producing enzyme: glucose-6-phosphate dehydrogenase (G6PD) (Zhou et al., 2016). In conclusion, Kglu may reduce or inactivate the activity of the enzyme, and the amount of NADPH will decrease, thus reducing the antioxidant defense ability of the nervous system and leading to nervous system damage. However, this still needs to be further verified.

Chen et al. (2020) found that mitochondrial ROS can cause endothelial dysfunction and hypertension. Therefore, oxidative stress is not only related to the nervous system but may also be an important mechanism of stress-induced cardiovascular disease (CVD). CVD is the main cause of morbidity and mortality worldwide, and metabolic dysfunction is the basic core mechanism of CVDs. Protein acylation plays an important role in the physiological and pathological processes of the heart and blood vessels (Chen et al., 2020). For example, malonylation can damage the activity of mTORC1 kinase and ultimately lead to angiogenesis defects, which is an important part of myocardial infarction (Bruning et al., 2018). Although the current studies on the relationship between Kglu and myocardial damage are less than that of malonylation, some important results have been discovered in recent years. For example, it’s found that the serum protein Kglu level decreased after acute myocardial infarction (Zhou et al., 2020). In addition, Zhou et al. (2016) found that SIRT5 KO mice showed higher sensitivity to cardiac ischemia-reperfusion injury, which was related to the increased production of ROS. Relevant experiments have confirmed that by reducing ROS production and alleviating mitochondrial swelling, the damage caused by mitochondrial membrane potential in mice’s cardiovascular system can be partially saved (Nagao et al., 2016; Yu et al., 2018). The association between Kglu and CVD is a new research field. In the future, it may be possible to reduce the oxidative stress after myocardial infarction by regulating the enzymes of Kglu, thereby reducing the damage to the heart.

### Regulation of Glutaric Aciduria Type 1

Glutariduria type 1 (GA1), a type of organic aciduria (Mahoney, 1976), was first reported by Goodman and Kohlhoff (1975). It is an autosomal recessive genetic disease that causes lysine and tryptophan metabolism disorders due to insufficient GCDH activity (Goodman et al., 1977). It is characterized by intermittent metabolic acidemia, dystonia, asthenia, and mental retardation (Stokke et al., 1976). The GCDH gene is located on human chromosome 19p13.2, spans about 7 kb, contains 11 exons and 10 introns (Goodman et al., 1977; Shadmehri et al., 2019), and it is involved in the degradation of L-lysine and L-tryptophan (Tan et al., 2014). It is known that GCDH degrades glutaryl-CoA, thereby reducing the Kglu level of the protein, and the GCDH deficiency will lead to the increase of glutaryl-CoA and Kglu (Tan et al., 2014).

Glutaric aciduria 1 is a multi-organ disease, and the organ most affected during metabolic abnormalities in patients is the brain (Zhou et al., 2016). GA1 is caused by a mutation in the gene of the mitochondrial stromal enzyme GCDH, which elevates the level of glutaric acid (GA) in the brain and blood (Koeller et al., 2002). GCDH deficiency could impair the degradation of lysine/tryptophan (Zhou et al., 2016), which may be an important reason for the increase of glutaric acid, because lysine/tryptophan is the source of glutaric acid. It is known that glutaric acid is one of the precursors of glutaryl-CoA, and the augment of glutaric acid will indirectly lead to the increase of glutarylation (Tan et al., 2014; Chen et al., 2020). Schmiesing et al. (2018) also believed that GCDH deficiency is related to the mitochondrial protein lysine Kglu in the pathogenesis of GA1 disease, which leads to the heterogeneity and fragility of glial cell mitochondria (Zhou et al., 2016). Is it possible to treat or alleviate the neurological symptoms of GA1 by regulating the protein Kglu? This is a challenging subject for researchers.

### Regulation of Chromatin Dynamics

Nucleosome is the basic repetitive unit of chromatin. Both structures are highly dynamic, and one main mechanism for controlling their dynamics is through PTMs of histone. H4K91 is a residue located at the interface between H3-H4 tetramer and H2A-H2B dimer (Bao et al., 2019). There are salt bridges between H4K91 and glutamate residues from histone H2B in nucleosome (Cosgrove et al., 2004).

**FIGURE 4:**
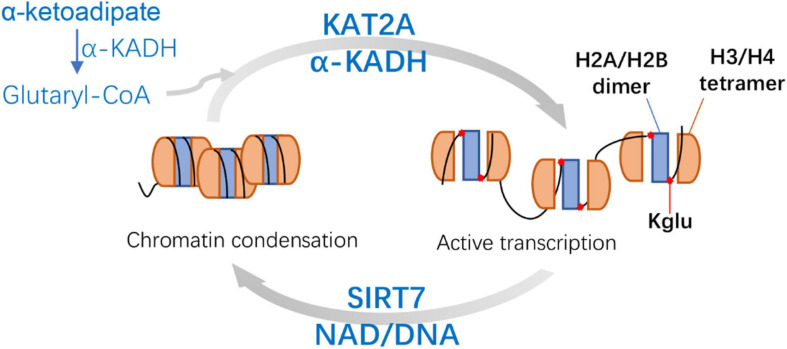
Mechanisms and features of Histone H4 Lysine 91 glutarylation (H4K91glu). KAT2A is coupled with α-KADH to catalyze the H4K91glu as the histone glutaryl transferase. H4K91glu could regulate chromatin structure and enhance active gene expression. SIRT7-catalyzed removal of H4K91glu is related to chromatin condensation. KAT2A, lysine acetyltransferase 2A; α-KADH, α-ketoadipate dehydrogenase; NAD, nicotinamide adenine dinucleotide.

It has been reported that a known histone acetyltransferase KAT2A (Grant et al., 1997; Wang and Dent, 2014) is coupled with α-ketoadipic dehydrogenase to catalyze the oxidative decarboxylation of α-ketoadipic acid to glutaryl-CoA as the histone glutaryl transferase (Bao et al., 2019). Then SIRT7 can catalyze the removal of H4K91glu. H4K91glu disturbs the efficient assembly of H2A/H2B dimer and H3/H4 tetramer to form octamer (Bao et al., 2019; Figure 4). Therefore, glutaryl groups of histones affect the stability of nucleosomes and chromatin. In mammalian cells, H4K91glu is mainly enriched in the promoter region of highly expressed genes. The downregulation of H4K91glu is closely associated with chromatin aggregation during mitosis and the response to DNA damage (Bao et al., 2019), suggesting that H4K91glu plays a vital role in modulating gene expression and chromatin damage. In addition, histone H4K91 was mutated into glutamate (K91E) in *S. cerevisiae* to simulate Kglu. The results consistently showed significant delays during S and G2/M phase in H4K91E mutant cells, suggesting that the K91E mutations (mimicking K91glu) may also destroy the assembly of nucleosome and chromatin during S phase and mitosis (Bao et al., 2019).

## Prediction of Glutarylation by Computational Tools

To better understand the molecular mechanism of Kglu, it is important to accurately identify the substrate of Kglu and its corresponding Kglu sites. The traditional method of identifying Kglu is based on affinity enrichment proteomics method (Chen et al., 2012): pan anti-Kglu antibody was used to enrich the glutarylated peptide, and then the HPLC-mass spectrometry (MS)/MS was used to analyze it (Chen et al., 2005). This experimental method is expensive, cumbersome and time-consuming (Xu et al., 2018). Therefore, some computational tools for predicting Kglu sites have been developed (Table 2). Based on known protein interaction data, using feature extraction and feature selection techniques (Chen et al., 2019), combined with probability theory and mathematical statistics, using recognized machine learning algorithms, such as support vector machines (SVMs) (Huang et al., 2019), random forest (RF) (Al-Barakati et al., 2019), to discover possible proteins Interaction site.

**TABLE 2:** Statistics of information on developed site prediction models.

**Time (year)**	**Tool**	**10-fold cross-validation**				**References**	**URL**
		**SN**	**SP**	**ACC**	**MCC**		
2018	GlutPred	65%	77%	75%	0.32	Ju and He, 2018	http://dx.doi.org/10.1016/j.ab
2018	iGlu-Lys	50%	95%	88%	0.51	Xu et al., 2018	http://app.aporc.org/iGlu-Lys/
2019	MDDGlutar	68%*	62%*	64%*	0.28*	Huang et al., 2019	http://csb.cse.yzu.edu.tw/MDDGlutar/
2019	RF-GlutarySite	81%	68%	75%	0.50	Al-Barakati et al., 2019	-
2020	PUL-GLU	72%	75%	75%	0.35	Ju and Wang, 2020	-
2020	BiPepGlut	70%	93%	82%	0.64	Arafat et al., 2020	www.brl.uiu.ac.bd/bioglutarylation
2021	iGlu_AdaBoost	87%	74%	80%	0.61	Dou et al., 2021	-

SN: sensitivity; SP: specificity; ACC: accuracy; MCC: matthew correlation coefficient. *5-fold cross-validation.

Ju and He (2018) discovered that kspaced amino acid pair features play an important role in the prediction of glutarylation sites. Then they established a predictive model GlutPred based on comprehensive features composed of amino acid factor (AAF), binary code (BE), and composition of k-spaced Amino Acid Pairs (CKSAAP). In the same year, Xu et al. (2018) used the characteristics of the position-specific propensity matrices (PSPM) to build a model iGlu-Lys, which improved the prediction performance. Huang et al. (2019) later developed a model MDDGlutar based on SVM, which combines six motifs identified by maximal dependence decomposition (MDD). This model significantly improves the predictive performance of Kglu sites recognition and takes into account sensitivity and specificity. Then Al-Barakati et al. (2019) used Random Forest (RF) to predict the Kglu sites from the primary amino acid sequence and established the model RF-GlutarySite. In terms of performance indicators that are most affected by TP rate (such as SN, PR, and F1 scores), RF-GlutarySite is superior to the existing glutaric acid site predictors. On the contrary, for indicators that are more sensitive to the TN rate (such as SP and ACC), it does not work well. Ju and Wang (2020) regarded the experimentally verified glutaric acid sites as positive samples, and the remaining unverified lysine sites as unlabeled samples. A new type of glutaric acid site predictor PUL-GLU was developed by using positive unlabeled (PU) learning technology. Based on the evolutionary characteristics of double peptides, Arafat et al. (2020) used Extra-Trees (ET) classifier to build the model BiPepGlut. Recently, Dou et al. (2021) believed that the physical and chemical properties of charge, polarity, and van der Waals volume play a key role in the recognition of protein glutarylation, especially the positively charged R and K residues around the Kglu sites. They used the ensemble classifier AdaBoost to identify Kglu sites and built a new computational predictor called iGlu_AdaBoost. Here is a comparison of these predictors. iGlu-Lys did not utilize the secondary structure and tertiary structure characteristics of protein, and not balance positive and negative data. The SN of iGlu-Lys is the lowest (Xu et al., 2018). GlutPred used a biased SVM algorithm to handle the unbalanced problem in the prediction of glutarylation sites and showed good performance in specificity (SP) and accuracy (ACC). However, there was a large gap between the positive and negative prediction abilities (Ju and He, 2018). The prediction results of iGlu-Lys and GlutPred were significantly biased toward the majority of samples (i.e., non-glutarylation sites), and the prediction efficiency of positive samples was lower (Dou et al., 2021). RF-GlutarySite helps discover the relationship between glutarylation and well-known lysine modifications, such as acetylation, methylation, and some recently identified lysine modifications. PUL-GLU could predict more non-glutaryl lysine sites (Al-Barakati et al., 2019). iGlu_AdaBoost has good prediction generalization ability, and the prediction results have high consistency between positive samples and negative samples (Dou et al., 2021).

From GlutPred to iGlu_AdaBoost, which was recently developed (not yet online), there are currently seven computational prediction models (Table 2). Besides, the number of positive and negative samples in the training and testing data sets were shown in Table 3. These tools provide researchers an easy way to discover new Kglu sites and proteins.

**TABLE 3:** The number of positive and negative samples in the training and testing data sets.

**Time (year)**	**Tool**	**Testing data set**		**Training data set**	
		**Positive**	**Negative**	**Positive**	**Negative**
2018	GlutPred	56	428	590	3498
2018	iGlu-Lys	-	-	-	-
2019	MDDGlutar	46	92	430	860
2019	RF-GlutarySite	44	203	400	400
2020	PUL-GLU	56	428	590	3498
2020	BiPepGlut	217	192	1952	1731
2021	iGlu_AdaBoost	44	203	400	1703

*-, Data are not available.*

## Conclusion

Although the study of Kglu of biological proteins started late, the research on Kglu is increasing quickly and has achieved great results. The development of high-resolution LC-MS/MS methods has made it possible for the identification of massive Kglu proteins. Kglu is involved in various pathways that control diverse cellular functions ranging from mitochondria to chromosomal histones. Current studies mainly focus on mitochondrial metabolism and related content (Tan et al., 2014; Schmiesing et al., 2018). The emergence of Kglu site prediction tools also accelerates the discovery of new Kglu sites.

However, some limitations in the previous reported studies still need to be addressed. One important question is the reasons for the small number of sites found in eukaryotes. We consider the following four aspects: (1) The antibody specificity. Although eight studies stated that their antibodies were highly specific and verified, the relevant data about the number of glutarylated peptides vs. unmodified peptides were not easily accessed from three studies. Hence, it is better to firstly collect these antibodies to evaluate their specificity in the same species/tissues, and then use the antibody with highest specificity to identify the glutarylated protein and sites in the new species/tissues. (2) The low stoichiometry of this modification (Schmiesing et al., 2018). It is well known that discovering a new, unknown PTM with low stoichiometry is a great challenge for analytical techniques. (3) The sample preparation. Different preparation methods will influence the purity of the sample, which in turn affects the specific binding of antibodies. During the sample preparation, if deglutarylase inhibitors are used in advance to inhibit deglutarylation, the number of identified glutarylated protein and sites will be increased. For example, Tan et al. (2014) identified more glutarylated proteins and sites from Sirt5^–/–^ mice, which can block deglutarylation with the deletion of SIRT5, and maximize the number of sites in tissues. But in other studies, these did not use inhibitors to block deglutarylation during sample preparation, which may reduce the number of sites (Schmiesing et al., 2018; Bao et al., 2019; Zhou et al., 2020). (4) The lability of this PTM. Kglu sites are characterized by instability and low abundance *in vivo* (Huang et al., 2019). If this PTM is decomposed before detection, it will result in a small number of sites found in eukaryotes. Hence, it is very important to add the stabilizer of PTM during sample preparation.

Another important question is the relationship between Kglu and cancer. As early as Tanaka et al. (1993) glutarylated the serum proteins of mice with glutaric anhydride, and found that glutarylation reduced the distribution of carrier protein in normal tissues, resulting in higher accumulation of tumor tissues. Therefore, they believed that glutaryl serum proteins have relative tumor selectivity and can be used as a macromolecular carrier for anti-tumor drugs, but this requires further research and verification. It is known that SIRT5 is responsible for de-glutarylation of Kglu (Tan et al., 2014). Recently, Osborne et al. (2016); Bringman-Rodenbarger et al. (2018), and Kumar and Lombard (2018) showed that SIRT5 plays an important role in cancer models, including tumor suppression and tumor metabolism (Osborne et al., 2016; Bringman-Rodenbarger et al., 2018; Kumar and Lombard, 2018). In addition, Carrico et al. (2018) believed that a potential key role of mitochondrial acylation in tumorigenesis is to initiate the Warburg effect. Hence, future studies are needed to uncover the role of Kglu in cancer.

For other questions about glutarylation, we have made a table (Table 4). For example, there are some Kglu sites that partially overlap with other PTMs such as Kac or Ksucc (Du et al., 2011; Chen et al., 2012; Park et al., 2013). Is there any crosstalk in the regulation of overlapping sites? Currently, most of the Kglu studies focus on the non-histone proteins, while the research on histone Kglu is relatively rare. Therefore, more research on histone Kglu is needed. As there are many problems, it is not necessary to list them all here, but these issues are basic and critical. We hope these questions can provide some enlightenment for future researchers.

**TABLE 4:** Scientific questions for future studies about Kglu.

**No.**	**Classification**	**Questions**
1	Mechanism	Is there any relationship between the regulation of Kglu, acetylation and succinylation overlap sites?
2		Does glutaryl transferase also exist in other parts such as the glutarylation of proteins in the cytoplasm and mitochondria?
3	Distribution	Why are there so few sites found in eukaryotes?
4		Is Kglu present in prokaryotes other than *E. coli* and *M. tuberculosis*?
5		Is there Kglu of prokaryotic biofilm proteins?
6	Function	Is it possible to treat asthenospermia and GA1 by regulating Kglu of proteins?
7		Is there any connection between Kglu and the development of cancer?
8		Except for H4K91, what is the function of other histone glutaric acid sites?

## Author Contributions

YX, FM, and LX conceived the study protocol, participated in the literature search and the data collection, analyzed the data, drafted the manuscript, and revised the manuscript. All authors read and approved the final manuscript.

## Conflict of Interest

The authors declare that the research was conducted in the absence of any commercial or financial relationships that could be construed as a potential conflict of interest.

## Footnotes

1 http://www.uniprot.org/docs/ptmlist

## Supplementary Material

Supplementary Table 1 The main methods and reagents used in previous researches.
