# Supplementary material for: Functions and Mechanisms of Lysine Glutarylation in Eukaryotes
Source: Front Cell Dev Biol. 2021 Jun 24;9:667684. doi: 10.3389/fcell.2021.667684 (PMC8264553; doi:10.3389/fcell.2021.667684)
Supplement: Supplementary Table 1 — The main methods and reagents used in previous researches. [file Data_Sheet_1.docx]

***Supplementary Material***

1. **Supplementary Table**

| Studies | Methods | Reagents | PubMed ID |
| --- | --- | --- | --- |
| Tan et al. | Western blotting;  Immunoprecipitation;  HPLC-MS/MS and MS | Anti-Kglu antibody; Anti-SIRT5 antibody; MS grade water; Acetonitrile; Recombinant HDACs | 24703693 |
| Xie et al. | LC-MS/MS Analysis;  Affinity Enrichment | Glutaryl-lysine antibody; Serum antibody; Trypsin NETN buffer; Trifluoroacetic acid (TFA) | 26903315 |
| Zhou et al. | Western blot;  Immunohistochemistry (IHC) analysis;  Immunoprecipitation; quantitative real-time PCR | Anti-SIRT5; Anti-PARP; Anti-caspase-3; Anti-pan-glutaryl-lysine (PTM-1151); H2O2; Paraquat; AntiFlag M2 affinity resin; Fluorescent dye 20,70-dichlorofluorescein diacetate (H2DCF-DA) | 27113762 |
| Schmiesing et al. | Immunoprecipitation;  Liquid Chromatography-MS;  Measurements;  MS Data Analysis;  IEF Gel Electrophoresis | Anti-Kglu antibody; Anti-GDH; Purified bovine glutamate dehydrogenase (GDH) RecombinantDLST; Electron transfer flavoprotein subunit B (ETFB); Glutaryl-CoA dehydrogenase (GCDH) Recombinant murine carbonic anhydrase 5B (Ca5b); Human carbonic anhydrase 4 (CA4) | 30208319 |
| Cheng et al. | Immunoblotting assay;  Indirect immunofluorescence assay | Pan-anti-glutaryllysine antibody (PTM-1151); Anti-P300 antibody (ab10485); Anti-glutaryl-CoA dehydrogenase (GCDH) antibody (ab112998); Anti-Sirtuin5 (SIRT5) antibody (15122-1-AP); Nicotinamide (NAM, purity: >98.0%); Human serum albumin solution (100 mg/mL, 10 064) | 31194865 |
| Bao et al. | Immunofluorescence;  Mass Spectrometry;  RNA sequencing analysis;  ChIP sequencing analysis;  Flow Cytometry;  Immunoprecipitation;  Non-Fluorophore-Labeled Nucleosome Reconstitution and Electrophoretic Mobility Gel Shift Assay (EMSA) | Antibodies specific to CPS1, H4K91glu, H3, H4, Sirt7, KAT2A and Sirt5.; Dynabead Protein A for Immunoprecipitation; Methyl methanesulfonate (MMS); RNase A; Proteinase K. | 31542297 |
| Zhou et al. | Immunoprecipitation;  MS Identification;  Affinity Enrichment | Anti–Sirt5 antibody (15122-1-AP); Anti-glutaryllysine antibody | 31532912 |
| Wang et al. | Quantitative real-time PCR (qPCR);  Western blot | HRP-conjugated goat anti-rabbit or anti-mouse IgG; Radio immunoprecipitation assay buffer Protease inhibitor cocktail; Phosphatase inhibitor cocktail; Trichostatin A; Nicotinamide | 32980686 |

**Supplementary Table 1.** The main methods and reagents used in previous researches.
